# A novel cold-chain free VCG-based subunit vaccine protects against *Chlamydia abortus*-induced neonatal mortality in a pregnant mouse model

**DOI:** 10.3389/fimmu.2023.1243743

**Published:** 2023-10-17

**Authors:** Shakyra Richardson, Courtnee R. Bell, Fnu Medhavi, Tayhlor Tanner, Stephanie Lundy, Yusuf Omosun, Joseph U. Igietseme, Francis O. Eko

**Affiliations:** ^1^ Department of Microbiology, Biochemistry and Immunology, Morehouse School of Medicine, Atlanta, GA, United States; ^2^ National Center for Emerging Zoonotic and Infectious Diseases, Center for Disease Control and Prevention (CDC), Atlanta, GA, United States

**Keywords:** *Chlamydia abortus*, Pmp18.3, vaccine delivery, protection, neonatal mortality

## Abstract

*Chlamydia abortus* (Cab) causes spontaneous abortion and neonatal mortality in infected ruminants and pregnant women. Most Cab infections are asymptomatic, although they can be treated with antibiotics, signifying that control of these infections may require alternative strategies, including the use of effective vaccines. However, the limitations imposed by live attenuated and inactivated vaccines further suggest that employment of subunit vaccines may need to be considered. The efficacy of a newly generated *Vibrio cholerae* ghost (rVCG)-based subunit vaccine harboring the N-terminal portion of the Cab Pmp18D protein (rVCG-Pmp18.3) in preventing Cab-induced abortion or neonatal mortality was evaluated in pregnant mice. Mice were intranasally (IN) immunized and boosted twice, 2 weeks apart with the vaccine, and immunized and unimmunized mice were caged with males 4 weeks postimmunization. The mice were then infected either IN or transcervically (TC) 10 days after pregnancy, and the fertility rate was determined 7 days postpartum. Eight days after delivery, the mice were sacrificed, and Cab infectivity in the lungs and spleens was evaluated by culturing tissue homogenates in tissue culture. Our results demonstrated that the vaccine induced immune effectors that mediated complete clearance of infection in the lungs and significantly reduced Cab infectivity in the spleen following IN immunization. Vaccine immunization also afforded protection against Cab-induced upper genital tract pathology (uterine dilation). Furthermore, while there was no incidence of abortion in both immunized and unimmunized mice, immunized mice were completely protected against neonatal mortality compared to unimmunized infected controls, which lost a significant percentage of their litter 7 days postpartum. Our results establish the capability of the rVCG-Pmp18.3 vaccine to prevent infection in the lungs (mucosal) and spleen (systemic) and protect mice from Cab-induced tubal pathologies and neonatal mortality, a hallmark of Cab infection in ruminants. To advance the commercial potential of this vaccine, future studies will optimize the antigen dose and the number of vaccine doses required for protection of ruminants.

## Introduction

The obligate intracellular Gram-negative bacterium, *Chlamydia abortus*, has affinity for the placenta of both animals and humans resulting in tissue damage, inflammation, preterm labor, still birth, and late-term spontaneous abortion (1, 2). Often, infected animals are asymptomatic until late pregnancy, and in sheep, abortion usually occurs approximately 2–3 weeks before delivery (3). Pregnant women may experience flu-like symptoms, which makes it difficult to diagnose without additional background knowledge of the patients’ contact with farm animals, leading to delayed antibiotic treatment, which may result in fetal death (4–6). However, while antibiotics are effective against *C. abortus* infection, most infections are asymptomatic, and so infected animals remain untreated. Thus, the use of an effective vaccine is necessary to control infection and limit its zoonotic potential.

While the current live attenuated vaccines derived from Cab strain 1B were thought to prevent infection, they have been shown to contribute to abortion storms in naive flocks, raising questions regarding their attenuation (7). In addition to being hazardous to produce, the vaccines are costly and difficult to produce in large amounts. Furthermore, during implementation of these vaccines, vaccination practices cannot easily be monitored, as it is difficult to distinguish infected from vaccinated animals (DIVA) using current serodiagnostic methods (8), which is possible with subunit vaccines (9). Inactivated whole organism-based vaccines have also played a role in managing disease in sheep in Europe, but their use is limited due to lack of protection against post-parturition bacterial shedding (10). These limitations motivated the increased efforts to develop safer efficacious vaccines capable of reducing chlamydial shedding and disease-associated reproductive failures, such as subunit vaccines. Despite the above challenges, no commercial *C. abortus* subunit vaccine is currently available.

Among the potential *C. abortus* proteins predicted and/or tested as vaccine antigens include the polymorphic membrane proteins (Pmps) that are like the type V autotransporter secretion system (11, 12). In addition to playing a role in chlamydial pathogenesis, the Pmps are expressed during all stages of the chlamydial life cycle and have been reported to contain immunogenic epitopes (13), stimulate innate immune responses (14), and therefore potential vaccine targets. We previously reported that a subunit vaccine candidate consisting of the N-terminal region of the polymorphic membrane protein (Pmp) 18D and the VCG delivery platform protected mice from *C. abortus* genital infection (15). However, this study did not evaluate protection against infertility, abortion, or neonatal death following challenge. We have redesigned this subunit vaccine to comprise T- and B-cell epitopes of the N-terminal region of the *C. abortus* Pmp18D and designated it Pmp18.3. In this study, we evaluated the efficacy of the newly generated subunit vaccine antigen expressed in *Vibrio cholerae* ghosts (rVCG-Pmp18.3) in preventing Cab-induced abortion or neonatal mortality in pregnant mice. Our results demonstrated that the vaccine completely protected mice from Cab-induced neonatal mortality and upper genital tract pathology compared to unimmunized infected control mice, which lost a significant percentage of their litter 7 days postpartum. Furthermore, the induced immune effectors mediated complete clearance of infection in the lungs and significantly reduced Cab infectivity in the spleen in comparison with unimmunized infected control mice.

## Materials and methods

### Ethics statement

This study was carried out in strict accordance with the recommendations in the Guide for the Care and Use of Laboratory Animals of the National Institute of Health. The Institutional Animal Care and Use Committee (IACUC) of Morehouse School of Medicine (MSM) approved the study protocol (Protocol Number 19-09). Six-week-old female C57BL6/J mice (stock number 000664) (The Jackson Laboratory, Bar Harbor, ME) were used in this study. Mice were allowed to acclimate for 3 days in the MSM Center for Laboratory Animal Resources (CLAR) facility prior to experimentation. All immunizations and challenge were performed under isoflurane anesthesia.

### Chlamydia stocks and antigens

Stock preparations of *C. abortus* AB7 organisms used in this study were generously provided by Dr. Teresa Garcia-Seco (VISAVET, Universidad Complutense de Madrid, Spain) and were titrated on Buffalo Green Monkey Kidney (BGMK) cell monolayers, and chlamydial elementary bodies (EBs) were purified by renografin density gradient centrifugation (Virusys Corporation, Randallstown, MD). *C. abortus* (Cab) antigen was prepared by exposing purified EBs to UV irradiation for 3 h and kept at −80°C pending use.

### Production of the rVCG-Pmp18.3 vaccine

The rVCG-Pmp18.3 vaccine candidate was generated by genetically inactivating *V. cholerae* cells harboring plasmid *pPmp18.3* as described previously (16). Briefly, *V. cholerae* O1 strain V912 cells co-harboring plasmid *pPmp18.3* coding for Pmp18.3 and the lysis plasmid pDKLO1 were cultured to mid-log phase in Brain Heart Infusion broth at 37°C, and rVCG-Pmp18.3 was generated by gene *E*-mediated lysis. At the end of lysis, harvested cells (rVCG preparations) were washed in a low ionic buffer before being lyophilized and stored at room temperature or refrigerated until use. In previous studies, rVCG preparations stored at room temperature maintained the stability of expressed antigens and the protective efficacy of vaccine formulations (15, 17–19).

### Immunization, challenge, and analysis of protection against Cab-induced abortion and neonatal mortality

Mice (10/group) were immunized intranasally (IN) with 1.5 mg of lyophilized rVCG-Pmp18.3 in 15 µl of PBS on days 0, 14, and 28 or were unimmunized (naive controls). Four weeks after the last immunization, immunized and unimmunized (nave) mice were mated with proven breeder males and weighed every 2 days for weight changes. Mice were also observed for vaginal plugs. If no plug was observed, any mouse that gained > 1.5 g over a 2-day period was considered pregnant. On day 10 of pregnancy, mice were challenged IN with 1 × 10^7^ or TC with 1 × 10^6^ IFUs of Cab strain AB7 in 5 ml of sucrose phosphate glutamic acid (SPG) buffer. Naive uninfected pregnant mice served as controls. Mice were observed for incidence of abortion, but not disturbed following challenge. Following delivery, the number of pups was recorded, and the mothers and pups were monitored and subsequently sacrificed on day 8. Unimmunized mice infected IN or TC between days 10 and 13 of pregnancy and similarly monitored served as infection only control group, while naive unimmunized mice mated with proven breeder males and monitored as above served as unimmunized controls.

### Determination of antigen-specific mucosal and systemic humoral immune responses

Serum and vaginal wash samples were collected from immunized and naive mice at 2 and 4 weeks postimmunization and kept frozen at −80°C until analyzed. The magnitude of Cab-specific IgG2c and IgA antibodies in the samples was quantified using a previously described ELISA technique (20). Briefly, 96-well plates (Costar) were coated in triplicate overnight with either 10 μg/ml of Cab antigen or IgA or IgG2c standards (0.0, 12.5, 25, 50. 100, 250, 500, and 1,000 ng/ml) to generate a standard calibration curve in 100 ml of PBS at 4°C. The results, which were simultaneously generated with the standard curve, show data as mean concentrations (ng/ml) ± SD and denote the mean of triplicates values for each assay.

### Determination of serum antibody avidity index

To evaluate the functionality of the anti-Cab IgG and IgG2c antibodies in serum samples obtained 2 and 4 weeks postimmunization, the avidity, or functional affinity, the total binding strength of the interaction between an antibody and antigen (21, 22) was evaluated using a modification of a previously described antibody ELISA assay (23) and the chaotropic agent, ammonium thiocyanate (NH_4_SCN). Thus, 96-well plates were coated overnight at 4°C with 10 μg/ml of Cab antigen. After washing with PBS containing 0.05% Tween 20 (PBS-T), non-specific binding sites were blocked by incubation with blocking buffer (PBS containing 5% goat serum and 1% BSA) for 1 h and then incubated with serum diluted 1:20 in blocking buffer at 37°C for 2 h. Following another wash, the wells were treated with 1M NH_4_SCN for 20 min at 37°C and incubated with Horseradish peroxidase (HRP)-conjugated goat anti-mouse IgG or IgG2c antibody (Southern Biotechnology, Birmingham, AL) for 1 h at 37°C. Plates were developed with the peroxidase substrate, 2,2'-azino-bis (3-ethylbenzthiazoline-6-sulfonic acid) (ABTS), and the absorbance was read at 450 nm. The relative avidity index was calculated by dividing the mean antibody concentration of samples in the presence of NH_4_SCN by the mean antibody concentration of samples in its absence. The relative avidity index was assessed based on the following criterion: values >50% indicate high avidity, between 30% and 49% indicate intermediate avidity, and those below 29% signify low avidity (24).

### Determination of *C. abortus* burden in mouse spleen

Eight days after parturition, spleens were harvested, weighed, homogenized using the gentleMACs Dissociator (Miltenyi Biotech, Gaithersburg, MD), and processed following the protocol described previously (25). Briefly, homogenates were diluted in PBS containing 2 mg/ml of DEAE dextran and centrifuged at 300×*g* for 10 min at 4°C. Approximately 25 µl of the supernatant diluted 1:1,000 in PBS DEAE dextran was added in triplicate to McCoy cell monolayers in a 96-well plate and incubated at 37°C for 2 h followed by 30 min of centrifugation at 1,400×*g*. The samples were replaced with 200 µl of complete Eagle’s Minimum Essential Medium (EMEM) supplemented with 10% FBS, 0.4% glucose, 0.01% sodium pyruvate, 0.1% yeast extract, 50 mg/ml of gentamicin, 2.5 mg/ml of amphotericin B, and 200 nM of glutamine, and incubated at 37°C, 5% CO_2_. After 48 h, the cells were fixed with 95% ethanol and stained with Remel™ PathoDx™ *Chlamydia* Culture Confirmation Kit (Thermo Fisher Scientific (Waltham, MA).

### Determination of *C. abortus* burden in mouse lungs

The lungs and livers harvested from immunized and control mice 8 days post-parturition were also weighed, and the lung tissues were homogenized using the gentleMACs Dissociator. Following centrifugation at 1,900×*g* for 30 min at 4°C, samples were vortexed for 30 s in 15-ml Falcon tubes containing glass beads, and supernatants (0.5 ml) were centrifuged at 2,000 rpm for 10 min at 4°C. Supernatants were diluted 1:1,000 in complete Iscove’s Modified Dulbecco’s Medium (IMDM) supplemented with 10% FBS and cycloheximide, and 200-µl aliquots were added in triplicates to confluent McCoy cell monolayers in 96-well plates. After centrifugation at 3,000 rpm for 1 h at 4°C and incubation for 2 h at 37°C in 5% CO_2_, the samples were replaced with 200 µl of fresh IMDM/cycloheximide and incubated further. After 48 h, the cells were fixed with 95% ethanol and stained with Remel™ PathoDx™ *Chlamydia* Culture Confirmation Kit (Thermo Fisher Scientific (Waltham, MA).

### Evaluation of protection against *C. abortus*-induced upper genital tract pathology following IN or TC challenge infection

Eight days postparturition, all study mice, including immunized and unimmunized mice challenged IN or TC, were euthanized, and the intact genital tracts were excised and examined *in situ* for gross pathological lesions.

## Results

### Induction of *C. abortus*-specific mucosal and systemic humoral immune responses

**Figure 1:**
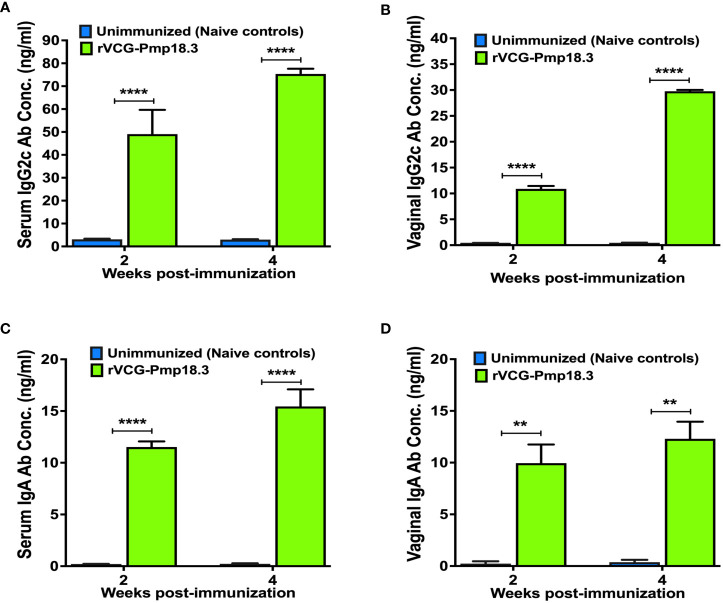
*Chlamydia abortus*-specific antibody responses elicited following IN immunization. Groups of mice were immunized intranasally, three times, 2 weeks apart. Unimmunized (naive) mice were included as controls. Serum and vaginal wash samples were obtained 2 and 4 weeks after the last immunization and pooled for each group. An antibody ELISA was used to determine the concentration of IgG2c and IgA in serum **(A, C)** and vaginal wash **(B, D)**. Results were generated simultaneously with a standard curve and display data sets corresponding to absorbance values as mean concentrations (ng/ml) ± SD of triplicate cultures for each experiment. The results are from two independent experiments with similar results, and the data shown are from one of the experiments. Significant differences between groups were determined by one-way ANOVA with Tukey’s post-multiple comparison test at p** <0.01 and p**** < 0.0001.

*C. abortus*-specific antibody responses elicited in serum samples and vaginal secretions collected at weeks 2 and 4 post-immunization were analyzed by an antibody ELISA assay. IN immunization with rVCG-Pmp18.3 elicited significantly higher (*p <*0.001) concentrations of Cab*-*specific IgG2c and IgA antibodies in both serum (**Figures 1A, C**) and vaginal secretions (**Figures 1B, D**) in comparison with unimmunized control mice. In addition, significantly higher (p< 0.05) amounts of IgG2c (**Figures 1A, B**) were observed in both serum and vaginal secretions compared to amounts of IgA (**Figures 1C, D**). The results indicate that in line with previous findings, IN immunization with rVCG-Pmp18.3 induces antigen-specific antibodies in both systemic and mucosal tissues, with IgG2c levels being consistently higher than IgA.

### IN immunization with rVCG-Pmp18.3 induced antigen-specific serum antibodies with high avidity

**Figure 2:**
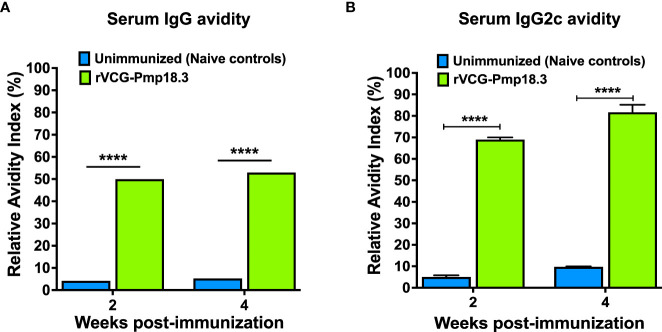
Relative Avidity Index of *Chlamydia*-specific serum IgG and IgG2c antibodies. Serum samples collected 2- and 4-weeks post-immunization were pooled, and the Relative Avidity was determined using a modified antibody ELISA assay in combination with the chaotropic agent, ammonium thiocyanate (NH4SCN). Samples diluted 1:20 were plated in triplicate, and the percent Relative Avidity Index (RAI) was calculated by dividing the concentration of treated samples by the concentration of untreated samples multiplied by 100. The data show the RAI values of serum IgG **(A)** and IgG2c **(B)** antibodies. Differences in the relative avidity between VCG-Pmp18.3 vaccine-immunized and unimmunized mice were compared by paired Student’s *t*-test at p**** <0.0001.

We hypothesized that the antigen-specific antibodies elicited in serum following immunization with rVCG-Pmp18.3 will have functional activity, indicated by high antigen binding capacity. Thus, the functionality of the Cab-specific IgG and IgG2c antibodies elicited in serum was established by assessing their relative avidity index using a modified antibody ELISA assay in combination with NH_4_SCN. The results presented in **Figure 2** show that while the relative avidity index values of serum IgG antibodies were moderate (**Figure 2A**), those of the Th1-associated IgG2c antibodies were high (>60%) 2- and 4-weeks post-immunization and increased with time; approximately 80% 4 weeks post-immunization (**Figure 2B**). On the other hand, the relative avidity index values of serum IgG and IgG2c antibodies from unimmunized control were at baseline levels at both timepoints (**Figures 2A, B**). The results indicate that the serum Th1-associated IgG2c antibodies show higher functionality compared to total IgG antibodies.

### IN immunization with rVCG-Pmp18.3 confers protection against infection in mucosal and systemic tissues following respiratory challenge

**Figure 3:**
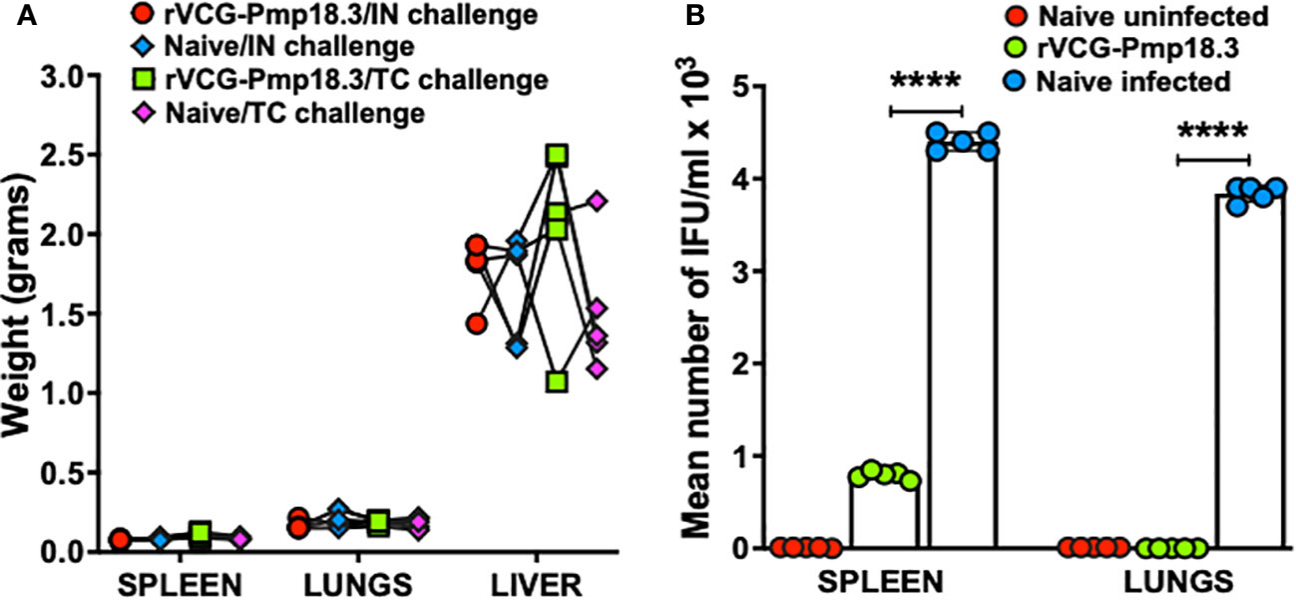
Protection against *C*. *abortus* infection in mucosal and systemic tissues following respiratory challenge. Groups of mice (n=5) immunized IN as described above were challenged intranasally with 1×10^6^ IFUs of live *C*. *abortus* AB7 at mid-pregnancy. Eight days after parturition, the harvested spleens, lungs, and livers were weighed prior to homogenization, and the mean weights (grams) per group was calculated. The homogenized spleen (systemic) and lung (mucosal) tissues were inoculated on McCoy cell monolayers, and the number of IFUs recovered from each mouse was enumerated. The data show the organ weights of **(A)** and the recoverable IFUs from each mouse **(B)** per group. The horizontal bars indicate the mean recoverable IFUs from each experimental group. Differences in the recoverable IFUs between the rVCG-Pmp18.3 vaccine immunized, and live *C*. *abortus*-infected control mice were compared by paired Student’s *t*-test at p < 0.05.

To assess vaccine efficacy against infection in mucosal and systemic tissues, rVCG-Pmp18.3- immunized and unimmunized mice were challenged intranasally with live *C. abortus* at mid pregnancy (day 10), and the bacterial burden in harvested spleens and lungs was determined 8 days postpartum. A comparison of organ weights between immunized and unimmunized mice challenged IN or TC revealed no significant differences (*p >*0.05) in the mean weights of the organs, irrespective of challenge route (**Figure 3A**). In addition, rVCG-Pmp18.3 vaccine-immunized mice completely cleared infection in the lungs (mucosal tissue) and had significantly (*p <*0.0001) reduced *C. abortus* burden in the spleen (systemic tissue) compared to the naive control mice, which showed high levels of *C. abortus* IFUs at this time point following IN challenge (**Figure 3B**). As expected, *C. abortus* was not isolated from the splenic and lung tissues of the naive uninfected control mice.

### IN immunization with rVCG-Pmp18.3 protects against neonatal mortality following challenge with *C. abortus* AB7

**Figure 4:**
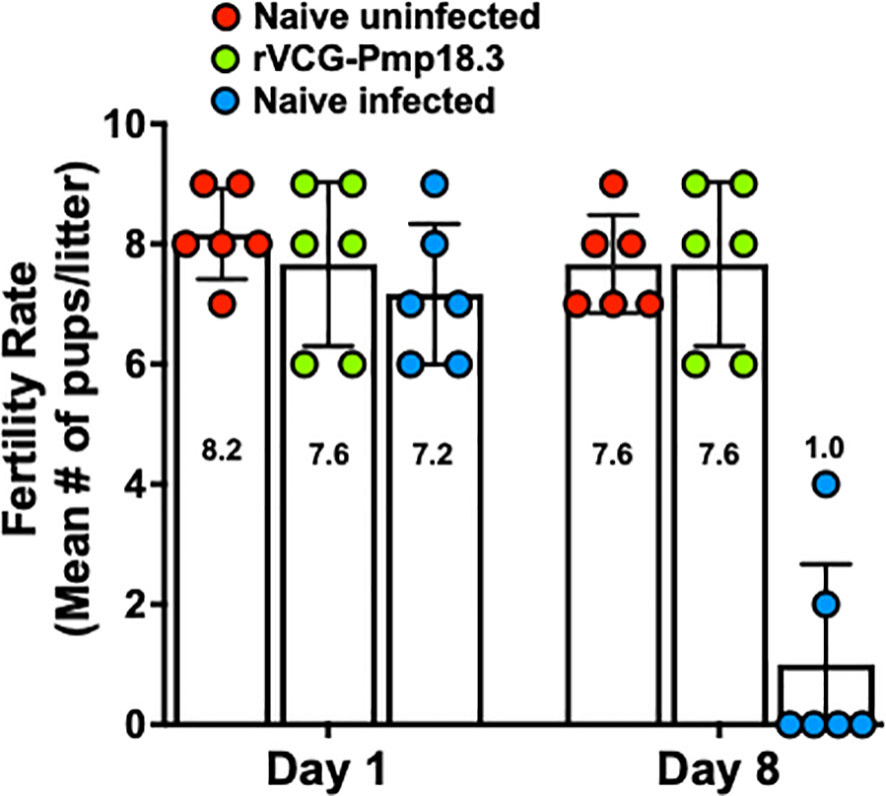
Protection against *C. abortus*-induced neonatal mortality by rVCG-Pmp18.3 vaccine following IN challenge with *C. abortus* AB7. Groups of mice (6/group) immunized IN with rVCG-Pmp18.3 vaccine as described above were mated with proven breeder males 3 weeks post-immunization and challenged IN with 1×10^6^ IFUs of live *C. abortus* on day 10 of pregnancy. Unimmunized infected and uninfected mice served as positive and negative controls, respectively. Each symbol represents a mouse in each group. The plotted data show the mean number of pups per mouse in each group on the day of birth (day 1) (fertility rate) and at the end of the observation period (day 8) post-parturition. Numbers within each bar represent the mean number of pups per liter for each experimental group. One-way ANOVA with Tukey’s post-multiple comparison test was used to determine significant differences between groups.

To determine vaccine efficacy against spontaneous abortion or neonatal death, rVCG-Pmp18.3 vaccine-immunized and unimmunized mice were challenged intranasally or transcervically with live *C. abortus* strain AB7 on day 10 of pregnancy and observed for spontaneous abortion. In addition, the fertility rates and pup survival were evaluated and compared with the naive uninfected group. The results show that neither the pregnant immunized nor the naive control mice aborted following IN infection. **Figure 4** shows the fertility rate (mean number of pups per litter) for each group on the day of delivery (day 1) and on day 8 following IN infection. The fertility rates of the rVCG-Pmp18.3 vaccine-immunized (7.6), naive infected (7.2), and naive uninfected control (8.2) mice were comparable on the day of delivery. However, 8 days after delivery, the fertility rate in the naive infected group (1.0) was significantly reduced with only two of six mice still having surviving pups compared to the vaccine-immunized and naive uninfected controls in which all six mice had surviving pups. The total number of pups per group on days 1 and 8 and the percent pup survival following IN infection of pregnant mice are shown in **Table 1**. While the vaccine-immunized mice retained a high percentage of pup survival (100%) like the naive uninfected controls (93%), only 14% of the pups in the naive Cab-infected mice survived following IN challenge. The results indicate that immunization with the rVCG-Pmp18.3 vaccine confers protection against neonatal mortality in mice following IN challenge infection.

**Table 1:** Total number and percent pup survival following IN challenge of pregnant mice.

Group	Total # of pups Day 1	Total # of pups Day 8	Percent pup Survival
rVCG-Pmp18.3	46	46	100
Naïve infected	43	6	14
Naïve uninfected	49	46	93

**Figure 5:**
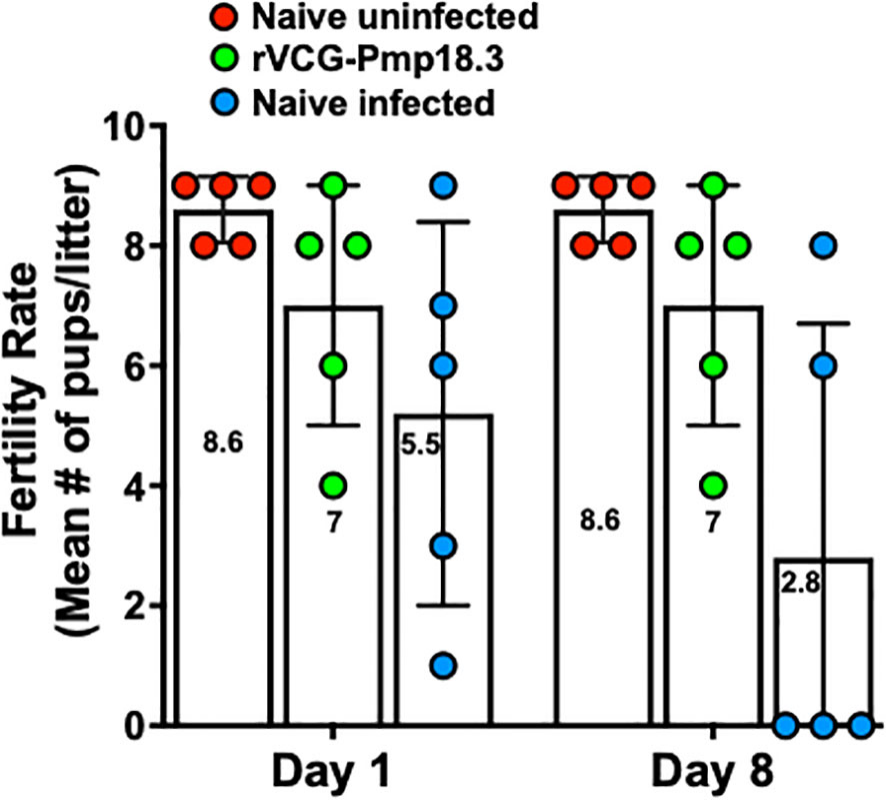
Protection against *C. abortus*-induced neonatal mortality by rVCG-Pmp18.3 vaccine following TC challenge with *C. abortus* AB7. Groups of mice (6/group) immunized IN with rVCG-Pmp18.3 vaccine as described above were mated with proven breeder males 3 weeks post-immunization and challenged TC with 1×10^6^ IFUs of live *C. abortus* on day 10 of pregnancy. Unimmunized infected and uninfected mice served as positive and negative controls, respectively. The plotted data show the mean number of pups per mouse in each group on the day of birth (day 1) (fertility rate) and at the end of the observation period (day 8) post-parturition. Each symbol represents a mouse in each group. Numbers within each bar represent the mean number of pups per liter for each experimental group. One-way ANOVA with Tukey’s post-multiple comparison test was used to determine significant differences between groups.

**Figure 6:**
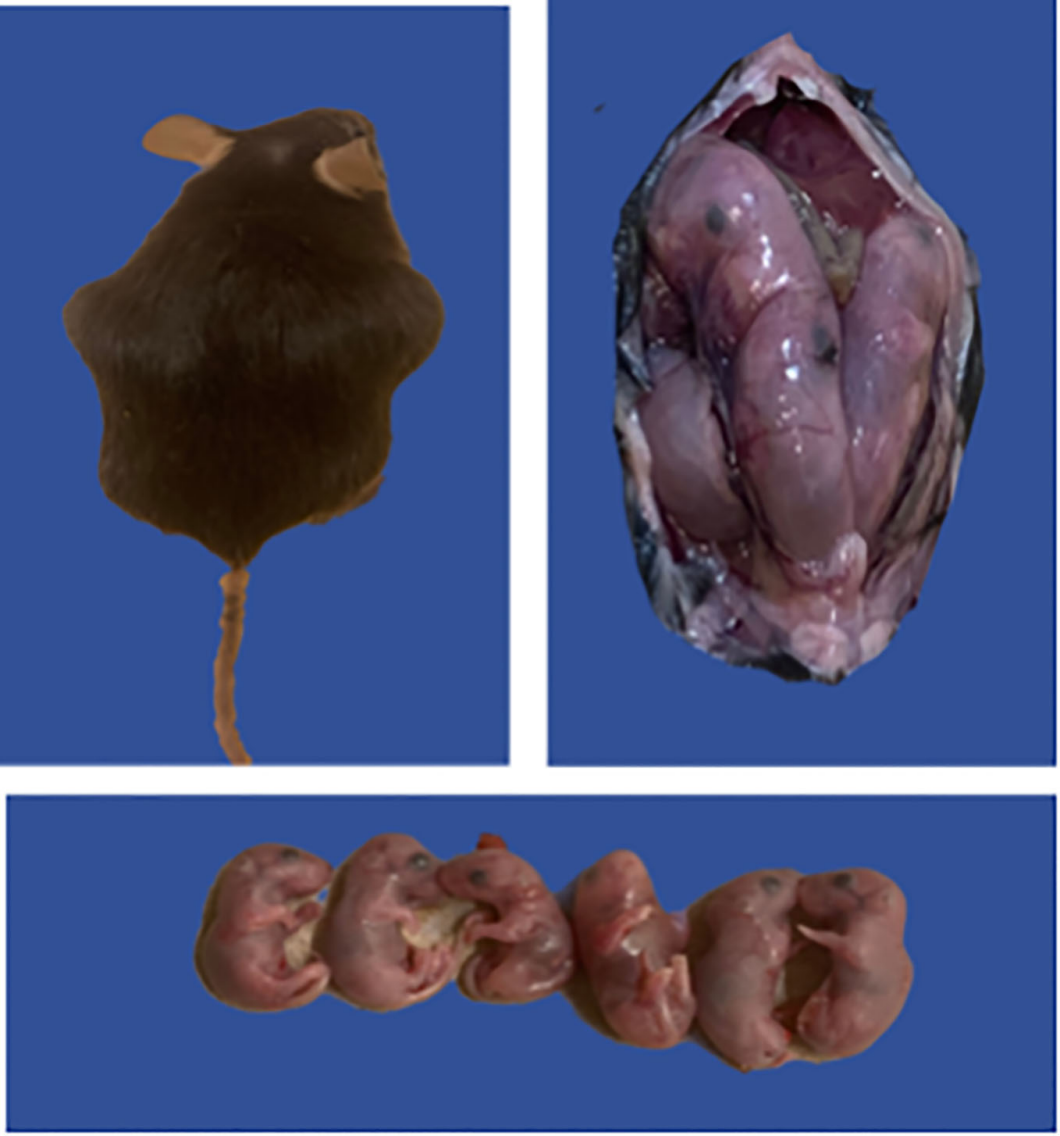
Possible case of *C. abortus*-induced dystocia and pre-term labor. Groups of mice (6/group) immunized IN with rVCG-Pmp18.3 vaccine as described above were mated with proven breeder males 3 weeks post-immunization and challenged TC with 1×10^6^ IFUs of live *C. abortus* on day 10 of pregnancy. Unimmunized infected and uninfected mice served as positive and negative controls, respectively. The figure shows one of the non-immunized TC infected control mice with an incidence of dystocia (difficult or obstructed labor); the mouse attempted labor 7 days post-infection and failed to deliver six pups.

We further investigated if challenge route affects the protective ability of the vaccine against neonatal death. Thus, IN immunized and unimmunized mice were challenged transcervically with live *C. abortus*, and the fertility rate and pup survival were evaluated as described above and compared with naive uninfected mice. **Figure 5** shows the fertility rate for each group of mice on the day of delivery (day 1) and on day 8 (the end of the observation period) following TC infection. Like the IN challenged mice, none of the pregnant immunized and naive control mice aborted following TC infection. The results also showed that the fertility rate of the vaccine-immunized/TC challenged (7.0) and the naive uninfected control (8.6) mice did not differ significantly on the day of delivery. However, like the findings with the IN challenged mice, the fertility rate in the naive/TC infected group 8 days after delivery was significantly reduced from 5.5 to 2.8 (**Figure 5**). **Table 2** shows the total number of pups per group on days 1 and 8 and the percent pup survival following TC infection of pregnant mice. The data show that whereas 53.8% of the pups in the naive TC infected mice survived, both the vaccine immunized/TC infected and naive uninfected control mice had 100% pup survival. The results indicate that route of infection has no impact on the protective efficacy of the rVCG-Pmp18.3 vaccine against neonatal mortality in mice following IN immunization. Furthermore, one of the non-immunized TC infected control mice revealed an incidence of dystocia (difficult or obstructed labor). The mouse was able to give birth to one single pup before labor was delayed due to improper positioning of the pups. Unfortunately, the single pup did not survive through the night. The mouse was observed for 24 h before being euthanized to prevent further distress, and six additional pups were found in the vaginal canal (**Figure 6**).

**Table 2:** Total number and percent pup survival following TC challenge of pregnant mice.

Group	Total # of pups Day 1	Total # of pups Day 8	Percent pup Survival
rVCG-Pmp18.3	35	35	100
rVCG-gD2 controls	26	14	53.8
Naïve uninfected	43	43	100

### IN immunization with rVCG-Pmp18.3 protects against *C. abortus*-induced upper genital tract pathology following IN or TC challenge infection

**Figure 7:**
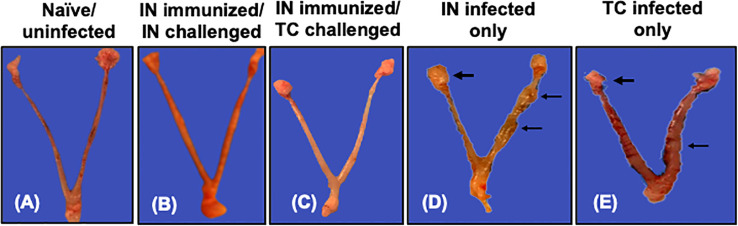
Protection against *C*. *abortus*-induced upper genital tract (UGT) pathology by rVCG-Pmp18.3 vaccine following IN or TC challenge infection. Groups of mice were immunized IN with rVCG-Pmp18.3 vaccine, mated with proven breeder males, and challenged IN or TC on day 10 of pregnancy as described above. Eight days after parturition, genital tracts were harvested from immunized and control mice and evaluated for the development of gross pathological lesions. Representative uterine horns of naive uninfected **(A)**, rVCG-Pmp18.3 vaccine-immunized/IN challenged **(B)**, and vaccine-immunized/TC challenged **(C)** mice with no evidence of visible pathology. Representative uterine horns of unimmunized/IN challenged **(D)** or TC challenged **(E)** control mice showing evidence of swollen inflamed oviducts (thick arrows) and tubal dilation with black nodular lesions (thin arrows).

The protective efficacy of the rVCG-Pmp18.3 vaccine against *C. abortus*-induced tubal pathology was evaluated by comparing the development of gross pathological lesions in the UGT of immunized and unimmunized mice challenged intranasally or transcervically on day 8 postpartum. As expected, no signs of gross pathological lesions were observed in the UGTs of unimmunized/uninfected control mice (**Figure 7A**). Interestingly, none of the genital tracts of mice challenged either IN or TC following IN immunization displayed any signs of pathological lesions (**Figures 7B, C**). In contrast, the genital tracts of unimmunized mice infected either IN (40%) or TC (80%) showed overt tubal dilation with black nodular lesions and dilated or swollen oviducts (**Figures 7D, E**). The results indicate that IN immunization with rVCG-Pmp18.3 protects against *C. abortus*-induced tubal pathology following IN or TC challenge.

## Discussion

*C. abortus* infection is a significant cause of still birth and late-term spontaneous abortion in sheep. Limitations and safety concerns of the current commercially available live attenuated vaccines derived from the 1B strain of *C. abortus*, which have been incriminated in disease causation in farm animals, have been reported (26). Moreover, although inactivated vaccines have been shown to reduce abortions in sheep in the UK, they fail to prevent bacterial shedding and reproductive failures (27). These observations have prompted the search for new vaccine candidates, including subunit vaccines that will in addition to being efficacious obviate the safety concerns and limitations imposed by live and inactivated vaccines. Reports indicate that ewes that have aborted following Cab infection develop strong protective immunity and are protected from preterm abortion in subsequent seasons (28), suggesting that subunit vaccines that mimic such immune responses could constitute safer and effective vaccines for ruminants. Although ruminants are the natural host for *C. abortus*, mouse models have served as viable precursors for immune response and vaccine efficacy studies because the pathogenesis of Cab infection in sheep and mice is similar (29). We previously reported that a subunit vaccine candidate based on the N-terminal portion of the *C. abortus* Pmp18D and VCG protected mice against genital *C. abortus* infection (15). However, with regard to protection against infertility, upper genital tract pathology, spontaneous abortion, or neonatal death, the hallmark of *C. abortus* (Cab) infection in ruminants was not investigated.

Thus, we tested the protective efficacy of a modified rVCG-based Cab subunit vaccine comprising T- and B-cell epitopes of the Cab Pmp18D (rVCG-Pmp18.3) against abortion or neonatal mortality after Cab genital challenge infection in pregnant mice. The possibility that cellular and humoral immune effectors may contribute to resolution of Cab infection prompted us to concurrently assess the specific cell-mediated and humoral immune responses in the serum and vaginal secretions of vaccine-immunized mice. Immunological evaluation revealed that rVCG-Pmp18.3 vaccine-immunized mice elicited significant *Chlamydia*-specific IgA and IgG2c antibody responses in serum and vaginal secretions. Although reports indicate that anti-Cab antibodies are detected in the serum of ewes after Cab infection (30), the role of antibody in protection against ovine enzootic abortion (OEA) is incompletely understood. It has previously been suggested that antibodies may contribute to protection against secondary infection either by blocking attachment of EB to target cells or opsonizing EBs (31). However, since the magnitude of antibodies does not directly correlate with antibody functionality, the quality of elicited antibodies was determined by assessing their relative avidity. Establishing the quality of elicited antibodies is important, since antibody function has been shown to correlate with post-vaccination protection against bacterial organisms by developing long-term B-cell memory responses (32) (33). The finding that rVCG-Pmp18.3 vaccine-immunized mice elicited high avidity serum IgG2c antibodies, which are associated with Th1-type responses that are required for successful vaccination against OEA (9), suggests that these antibodies possess high functional activity.

Unlike *C. trachomatis*, which is genitally acquired, acquisition of Cab is mainly via the respiratory route, and following infection, it is spread through the blood and lymph to several organs, including the lungs, liver, spleen, and the placenta (34–36). Vaccine efficacy analyses against respiratory challenge with *C. abortus* strain AB7 revealed a significant decrease in chlamydial burden in the spleens and complete protection against challenge infection in the lungs, demonstrating the efficacy of the vaccine against mucosal and systemic infection. Change in lung weight has been established as an indicator of the severity disease in murine models of respiratory infections (37, 38). There was no correlation between lung weights and disease severity. However, lung weights were only recorded at a one-time point (day 18 post-challenge), and lung weights did not differ significantly between immunized and control mice. Moreover, reports indicate that changes in organ weights correlated with disease severity in the initial phases of infection (38–40). Furthermore, 18 days post-challenge may have been too late to observe significant differences in organ weights. To minimize contact with the mice during pregnancy, vaginal swabs were not collected, and so vaccine efficacy against genital Cab infection was not evaluated in this study.

Since Cab infections can cause upper genital tract (UGT) pathology leading to infertility or abortion and neonatal mortality (41, 42), we evaluated the protective efficacy of the vaccine against *C. abortus*-induced abortion or neonatal mortality in a pregnant mouse challenge model following IN and TC infection. The transcervical route has been used as a non-surgical method to bypass the cervical blockade in mice to initiate infection leading to upper genital tract inflammation (43–45). Comparison of *C. abortus*-induced abortion or neonatal mortality between immunized and control mice on day 8 postpartum showed that neither the immunized nor the unimmunized control mice aborted following both IN and TC challenge. These findings in mice confirm previous reports indicating that infected pregnant ewes may deliver live weak lambs, which fail to survive, cited by (46). In addition to reduced pup survival, the unimmunized mice infected TC also showed a significant reduction in fertility rate compared to those infected IN. The explanation for this disparity is uncertain given that the respiratory route is the natural route of Cab infection in ruminants leading to preterm abortion and other reproductive complications (47). On the other hand, the finding that the IN-infection route resulted in a significantly higher incidence of pup mortality in naive infected mice compared to the TC route is consistent with previous reports indicating that the respiratory route is the route by which Cab infection occurs naturally (35, 36, 47–49). The complete protection afforded by the vaccine against Cab-induced neonatal mortality in immunized mice following IN and TC challenge strongly suggests its potential as an effective vaccine for use in ruminants. Another finding in this study was a single incidence of dystocia in the TC-infected mice. This mouse attempted delivery 7 days post-challenge, approximately 18 days post-pregnancy, and was only able to deliver one weak fetus, which did not survive for 24 h. It is unclear if the TC inoculation contributed to the occurrence of dystocia in this mouse. Although dystocia is common among laboratory mice (50), the occurrence of dystocia in combination with premature delivery attempt may suggest the involvement of Cab infection.

Cab infections also induce severe UGT pathology involving inflammation and fibrosis, often leading to tissue damage and hydrosalpinx (41, 42). The absence of any evidence of gross pathological lesions in all vaccine-immunized mice following IN (natural route of infection) or TC challenge contrasts with the overt tubal dilation with black nodular lesions and dilated or swollen oviducts observed in the UGT of unimmunized mice, with greater severity occurring in TC-infected mice. These results corroborate the outcomes of a previous study that reported the protective efficacy of a rVCG-based vaccine against *C. trachomatis*-induced UGT pathology, involving oviduct and uterine inflammation following rectal immunization and intravaginal challenge (18). Previous reports indicate that a shift in the balance of a Th1- to a Th2-type immune response that leads to a decrease in IFN-γ and an increase in IL-4 and IL-10 production, which antagonizes and downregulates Th1-type cytokines, allows the proliferation and colonization of the placenta by Cab, triggering abortion (47). Neutrophil depletion has been reported to lead to a reduction in IFN-γ levels (51), while CD8+ T cell depletion in the spleens of experimentally infected mice led to an increase in IFN-γ secretion, which limits Cab multiplication (52). Another significant finding in this report is the observation that UGT pathological lesions, indicated by dilated uterine horns and inflamed oviducts, correlated with neonatal mortality. However, although visible gross pathology can indicate pregnancy outcomes, it is not always certain, since delivery of live offspring does not indicate the absence of Cab infection (53).

In conclusion, our study demonstrates that IN immunization with the VCG-based Cab vaccine stimulated immune effectors that mediate Cab clearance in lung (mucosal) and splenic (systemic) tissues. The study also showed that immunization with the vaccine protected against Cab-induced neonatal mortality and tubal pathologies after IN and TC challenge. The complete protection afforded by the vaccine against Cab-induced respiratory mucosal infection, neonatal mortality, and tubal pathologies, a hallmark of Cab infection in ruminants, strongly suggests its potential as an effective vaccine for ruminants. To advance the commercial potential of this vaccine, future studies will optimize the antigen dose and the number of vaccine doses required for protection of ruminants.

## Data availability statement

The original contributions presented in the study are included in the article/supplementary material. Further inquiries can be directed to the corresponding author.

## Ethics statement

The animal study was approved by The Institutional Animal Care and Use Committee (IACUC), Morehouse School of Medicine (MSM) (Protocol Number: 19-09). The study was conducted in accordance with the local legislation and institutional requirements.

## Author contributions

FE conceived and designed the study. SR performed all the experiments. FM, TT, CB, and SL contributed to the experimental work. FE, SR, JI, and YO contributed to analysis and interpretation of data. SR and FE wrote the manuscript. All authors contributed to the article and approved the submitted version.
